# Cell-Free RNA as a Novel Biomarker for Response to Therapy in Head & Neck Cancer

**DOI:** 10.3389/fonc.2022.869108

**Published:** 2022-05-06

**Authors:** Anela Tosevska, Marco Morselli, Saroj K. Basak, Luis Avila, Parag Mehta, Marilene B. Wang, Eri S. Srivatsan, Matteo Pellegrini

**Affiliations:** ^1^ Department of Molecular, Cell and Developmental Biology, University of California at Los Angeles, Los Angeles, CA, United States; ^2^ Division of Rheumatology, Department of Medicine 3, Medical University of Vienna, Vienna, Austria; ^3^ Department of Surgery, Veterans Administration Greater Los Angeles Healthcare System, David Geffen School of Medicine, University of California at Los Angeles, Los Angeles, CA, United States; ^4^ Aveta Biomics Inc, Bedford, MA, United States; ^5^ Department of Head and Neck Surgery, David Geffen School of Medicine, University of California at Los Angeles, Los Angeles, CA, United States; ^6^ Jonsson Comprehensive Cancer Center, University of California at Los Angeles, Los Angeles, CA, United States; ^7^ Molecular Biology Institute, University of California at Los Angeles, Los Angeles, CA, United States

**Keywords:** liquid biopsy, cfRNA, head & neck, biomarkers, curcumin

## Abstract

Liquid biopsies are gaining more traction as non-invasive tools for the diagnosis and monitoring of cancer. In a new paradigm of cancer treatment, a synergistic botanical drug combination (APG-157) consisting of multiple molecules, is emerging as a new class of cancer therapeutics, targeting multiple pathways and providing a durable clinical response, wide therapeutic window and high level of safety. Monitoring the efficacy of such drugs involves assessing multiple molecules and cellular events simultaneously. We report, for the first time, a methodology that uses circulating plasma cell-free RNA (cfRNA) as a sensitive indicator of patient response upon drug treatment. Plasma was collected from six patients with head and neck cancer (HNC) and four healthy controls receiving three doses of 100 or 200 mg APG-157 or placebo through an oral mucosal route, before treatment and on multiple points post-dosing. Circulating cfRNA was extracted from plasma at 0-, 3- and 24-hours post-treatment, followed by RNA sequencing. We performed comparative analyses of the circulating transcriptome and were able to detect significant perturbation following APG-157 treatment. Transcripts associated with inflammatory response, leukocyte activation and cytokine were upregulated upon treatment with APG-157 in cancer patients, but not in healthy or placebo-treated patients. A platelet-related transcriptional signature could be detected in cancer patients but not in healthy individuals, indicating a platelet-centric pathway involved in the development of HNC. These results from a Phase 1 study are a proof of principle of the utility of cfRNAs as non-invasive circulating biomarkers for monitoring the efficacy of APG-157 in HNC.

## Introduction

Head and Neck (HN) cancer is a heterogeneous group of cancers in the oral cavity, pharynx, larynx, paranasal sinus, nasal cavity, or salivary glands, with squamous cell carcinoma being the major disease phenotype arising in the mucosal surfaces lining the aerodigestive tract (1, 2). Over 90% of Head and Neck Squamous Cell Cancer (HNSCC) cases are squamous cell cancers (2) and vast majority of them occur in the oral cavity and oropharynx (3, 4). Worldwide, an estimated 476,125 people are diagnosed with oral cavity and oropharyngeal cancers and 225,900 people die of the disease annually (5). American Cancer Society estimates 54,000 new incidence of and 11,230 deaths from oral cavity and oropharyngeal cancer in 2022 (6). Overall survival depends on many factors; two important factors are (i) underlying etiology of the disease, and (ii) the stage at initial diagnosis. For patients diagnosed early (stage I and II), with localized tumors without lymph node invasion and who are candidates for surgical resection with clear margins, the survival rates range from 50% to 90% according to the Surveillance, Epidemiology, and End Results (SEER) program of the National Cancer Institute (7). However, about two-thirds of patients are diagnosed at advanced stages (stage III and IV), where recurrence rates are as high as 50% within two years of the diagnosis of the primary tumor (8). For recurrent and metastatic patients with failure after first-line therapy have a median overall survival of about 1 year. Generally, most oral cavity cancers are Human Papilloma Virus (HPV) negative and most oropharynx cancers are HPV positive. A retrospective analysis (9) of 323 patients with advanced oropharyngeal squamous cell cancer enrolled in a randomized clinical trial found a 3-year overall survival rate of 82.4% for HPV-associated disease vs 57.1% for HPV-negative disease, and progression-free survival of 73.7% vs 43.4%. Even if the cancer is eliminated, survivors of head and neck cancer treatment may experience disability and morbidity due to the disease as well as the treatment. Patients with HNC, of which oral and oropharyngeal cancer make up the largest fraction, report significant and persistent physical (i.e., radionecrosis, mucositis, loss of taste, and dysphagia), functional (i.e., pain, difficulty swallowing, voice impairment, and poor dental status), and psychosocial problems (i.e., depression, disfigurement, social isolation, and delays returning to work) (10).

Results of cancer clinical studies suggest that single pathway inhibitors are unlikely to be the answer to controlling complex tumors, which have multiple molecular level aberrations (11). There is increasing recognition among cancer researchers that an approach that targets multiple pathways is needed, where in addition to inhibiting a single targeted pathway, there should be modulation of other complementary signaling pathways in order to develop effective treatments and prevent resistance and relapse while treating cancer. APG-157 is a synergistic composition of multiple molecules designed to modulate multiple proteins and transcription processes implicated in the pathogenesis and progression of Head and Neck Cancer. Results of a successful Phase 1 clinical evaluation of APG-157 have been reported (12). However, there is a need to understand the global results of simultaneously modulating multiple targets and study the impact of the drug at the cellular level. Typically, biomarkers for HNC used for prognostic evaluation include HPV positivity, p16 tumor suppressor protein expression and mutations of the p53 gene. Other biomarkers include mutations of PI3KCA and Notch1, and amplification of EGFR (epidermal growth factor receptor) seen in 15% of HNSCC (13, 14). These biomarkers often require invasive tumor tissue biopsy, which is impractical, especially when repeated sampling is required.

Attempts have been made to use non-invasive techniques to identify HNC biomarkers. Genomics, transcriptomics, and proteomics have been used to identify markers from salivary cells, and exosomes (15, 16). Salivary biomarkers, such as inflammatory cytokines, IL-6, IL-8 and TNFα are shown to be overexpressed in aggressive HNC, and salivary exosome markers such as miRNAs, miR-27b, 31, 125a, 139 and 200a (17) have been utilized for diagnostics. Circulating cell-free nucleic acids (NAs) have recently gained traction as minimally invasive biomarkers for disease detection, in particular cancers (18). In addition, circulating tumor cells (CTCs) and tumor DNA have been used in the context of cancer metastasis (19, 20), and have shown a great potential for disease diagnosis and prognosis in HNSCC (21–23), however, the isolation of these cells is technically challenging and requires a large amount of starting material for cell isolation. While cell-free DNA has been used extensively as biomarkers for multiple types of cancers, the use of cell-free RNA has been sparse. In addition, very few studies have focused on liquid biopsies in HNC, and they have used cfDNA or CTCs to identify putative biomarkers (24, 25). At present, only a single study has investigated the circulating transcriptome in HNC, focusing on lncRNAs (26), and identified HOXA11-AS, LINC00964 and MALAT1 as potential early circulating biomarkers of HNC. However, to our knowledge, no studies have shown the potential to utilize circulating RNAs as markers of treatment effectiveness in HNC. In the current study, we utilize a subset of samples from a double-blind placebo-controlled clinical trial of APG-157 on HNC patients (12) to test the hypothesis that cfRNAs can be utilized as biomarkers of HNC and treatment response.

## Materials and Methods

### Patients, Study Product, Treatment and Sample Collection

The patient population and study design and procedure have been described by Basak et. al (12). Briefly, subjects were recruited from the ear, nose, and throat clinics at the Veterans Administration Greater Los Angeles Healthcare System (VAGLAHS) in Los Angeles, California. Inclusion criteria were age >18 years, English fluency, and no history of prior chemotherapy or radiotherapy, or inflammatory conditions of the oral cavity or oropharynx. Cancer patients had biopsy-proven HNSCC. The study was conducted under IND 125454 and approved by the institutional review board (IRB PCC#2017-090885) of the VAGLAHS. Study recruitment and treatment protocols were approved by the VAGLAHS IRB. In this paper, we focused on a subset of 10 patients out of the 25 who completed the study.

The drug APG-157 is under clinical development by Aveta Biomics Inc. The product contains turmeric extract derived from the plant *Curcuma longa* with curcumin being the main component. APG-157 is encapsulated in a soft lozenge, containing 100 mg drug substance, which disintegrates slowly in the oral cavity over 15 to 20 minutes to release the substance. It is produced under current good manufacturing practice (cGMP) conditions to meet US Food and Drug Administration Chemistry, Manufacturing, and Controls guidance ensuring the consistency and quality of the pharmaceutical grade product.

For a schematic representation of the trial design see Basak et. al (12). Briefly, the drug APG-157 or placebo control gelatin pastilles, were administered. The drug was delivered transorally each hour for 3 consecutive hours for a 1-day treatment. A total of 25 subjects completed the study, out of which 10 were randomly sampled to be included in the current study: 4 control individuals (2 in the APG-157 treatment group and 2 in the placebo group) and 6 patients with oral cancer (3 in the APG-157 treatment group and 3 in the placebo group). Blood was collected before treatment and each hour after treatment (3 collections), and 1 sample was collected 24 hours after treatment. Samples collected before treatment, 3h post treatment and 24h post treatment were used for further analysis.

### RNA Extraction, Library Preparation and Sequencing

Collection and isolation of plasma using appropriate collection tubes have been described earlier (12). RNA was isolated from a maximum of 400uL plasma using the QIAGEN miRNeasy serum/plasma kit (catalog # 217184). RNA concentration and fragmentation were determined using TapeStation (Agilent Technologies) and sequencing libraries were prepared using the Ovation SoLo RNAseq kit (NuGEN, cat# 0500-32). Sequencing was carried out at the JCCC sequencing core facility using multiple Illumina sequencers as follows: NextSeq for batch 1, HiSeq3000/4000 batch 2 and NovaSeq for batches 3 and 4.

### Computational Analysis of the Transcriptome

Raw reads were quality checked using FastQC (27) and aligned to the human genome version GRCh38/hg38 using STAR v.2.4.1a (28). PCR duplicates were removed using UMItools (29) and features were quantified from mapped files using htseq-count (30). The intron-to-exon ratio was calculated using a custom script, and intron/exon gtf files were downloaded from the UCSC table browser (31). Data was imported and normalized in DESeq2 (32), and batch correction was performed using the *removeBatchEffect* function from the limma package (33). Sample distances calculation and PCA were performed using DESeq2 and R. Differential expression was performed using DESeq2 and differentially expressed genes were defined as genes with an absolute fold change larger than 2 and FDR-corrected p-value below 0.1. Overrepresentation analysis and GO term enrichment were performed using Metascape (34) and EnrichR (35). All analyses were performed using R v.3.6.1.

### Cell-Type Deconvolution Analysis

DESeq-normalized counts were used as input for the deconvolution analysis. Two publicly available computational tools, CIBERSORTx (36) and GEDIT (37) were used to estimate cell type proportions from cfRNAseq data, using the default parameters. Reference datasets that were used for the comparative analysis have been published previously (38, 39).

## Results

Characteristics of the dataset and the complete study design have been described in Basak et al (12). Here, we utilize a subset of the samples from Basak et al, as outlined in **Table 1**. The samples were processed in 4 batches with some minor differences as outlined in the methods.

**Table 1:** Characteristics of the study population.

Sample	Cancer	Placebo (mg)	APG 157 (mg)	Ethnicity	Gender	Age	Site	Stage	P16 expression	Smoking history
P1	Yes	NA	100	White	Male	66	Left tonsil	T2N1	Positive	Quit 1985
P2	No	100	NA	White	Male	34	–	–	–	Former occasionally
P3	Yes	100	NA	White	Male	68	Tongue	T2N0	Negative	No
P4	No	200	NA	Black	Male	49	–	–	–	1/2 PPD
P5	No	NA	200	Black	Male	56	–	–	–	No
P6	No	NA	200	Black	Male	55	–	–	–	1PPD
P7	Yes	NA	200	Black	Male	46	Hypopharynx	T4N2b	Negative	Quit 1985
P8	Yes	200	NA	White	Male	70	FOM	T2N2b	Negative	1PPD
P9	Yes	200	NA	White	Male	62	Right tonsil	T3N2b	Positive	1/2PPD
P10	Yes	NA	200	White	Male	64	FOM	T4aN2a	ND	2 PPD

NA, not applicable; ND, no data available; FOM, floor of mouth; PPD, packs per day; “-” healthy subjects without cancer.

### Batch Effects and RNA Fragmentation

**Figure 1:**
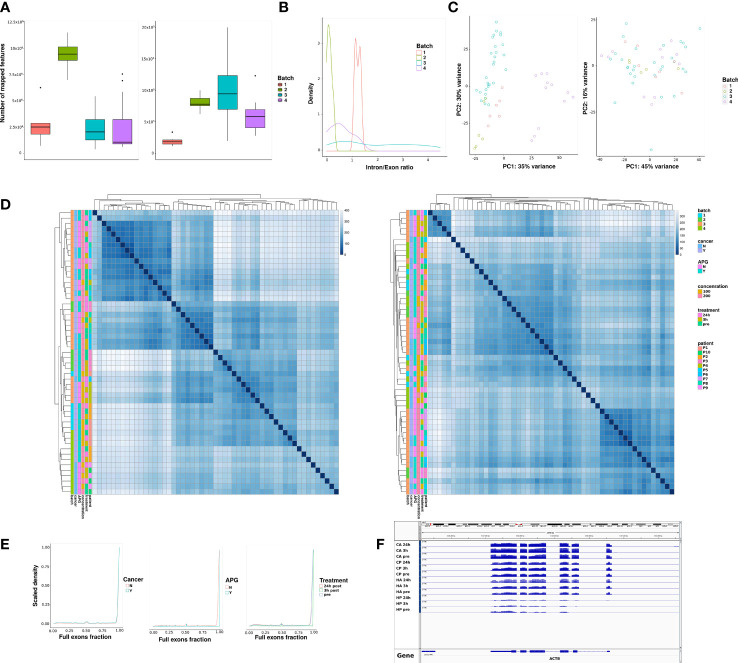
Characteristics of the sequencing runs. Samples were run in four batches under different conditions. **(A)** Number of sequencing reads mapping to features of interest (exons) per sequencing batch, non-corrected (left), after mitochondrial transcript removal (right). **(B)** Intron to exon ratio per sequencing batch. **(C)** Principal component analysis after normalization and variance-stabilizing transformation of samples before (left) and after batch correction (right). **(D)** Distance matrices between samples before (left) and after (right) batch correction. Each row and column represent a single sample, and the diagonal represents samples that are identical. A higher distance score represents higher dissimilarity between a pair of samples. Sample annotation is shown by multiple variables, such as: batch, whether a patient has cancer or not, whether they received 100mg or 200mg of APG-157 or Placebo, treatment time point and patient ID. We have included the same batch and cross-batch technical replicates for a portion of the samples. **(E)** The distribution of exon occupancy across all genes with 2 or more exons; a value of 1 means full occupancy; a value between 0 and 1 means partial exon occupancy; genes with no exon occupancy have been removed from the dataset. **(F)** An IGV screenshot showing the coverage of ACTB as a representative gene with full exon occupancy.

In order to estimate the influence of different RNA extraction/RNA sequencing batches we performed the experiments in four different sequencing runs. Runs 2 and 3 contained technical replicates using two extractions per sample. Batch 1 and 4 contained technical replicates from two patient samples in order to estimate the concordance between runs. **Figure 1A** shows the distribution of mapped reads per sequencing run that mapped to exons in the human genome. There was an uneven number of mapped features in batch 2, much of it influenced by an increased percentage of mitochondrial transcripts. The right panel of **Figure 1A** shows the feature count distribution after removing transcripts originating from the mitochondria. These data were used for subsequent analysis.

In order to estimate potential DNA contamination in any of the batches, we calculated the ratio of reads originating from intronic vs. exonic regions. **Figure 1B** shows the distribution of intron-to-exon ratio scores per batch. While the ratios differed slightly between batches, there was no indication of DNA contamination in any of the samples.

Principal component analysis (PCA) revealed clear global differences between sequencing batches (**Figures 1C, D**, left panels). These differences could be compensated by employing a batch correction step (**Figures 1C, D**, right panels). While this step might slightly over-correct and minimize the biological variance, and based on the results obtained from the PCA we found that including the batch term in downstream differential expression analysis models was appropriate for our study design.

We also set out to estimate the extent of RNA fragmentation in our data. As cell-free DNA is highly fragmented we were interested in estimating the exon occupancy for detected mRNAs with at least two constitutive exons. As seen in **Figure 1E**, the majority of detected RNAs showed full exon occupancy, meaning all of the exons were detected in plasma. A small peak at about 50% indicated that some RNAs (potentially ones having two constitutive exons) have only 50% of exons detectable in plasma. We could further confirm that there was no difference in exon occupancy in plasma cfRNA between patients and samples. **Figure 1F** shows one example of a gene, Actin-beta (ACTB), that can be fully detected in plasma of all the patients, albeit at different levels. This could either indicate that we are able to detect full-length transcripts in circulating plasma, potentially associated with extracellular vesicles, or that despite fragmentation, mRNAs can be “reconstructed” in patient plasma following sequencing.

### Cell Type Deconvolution From Plasma cfRNA

**Figure 2:**
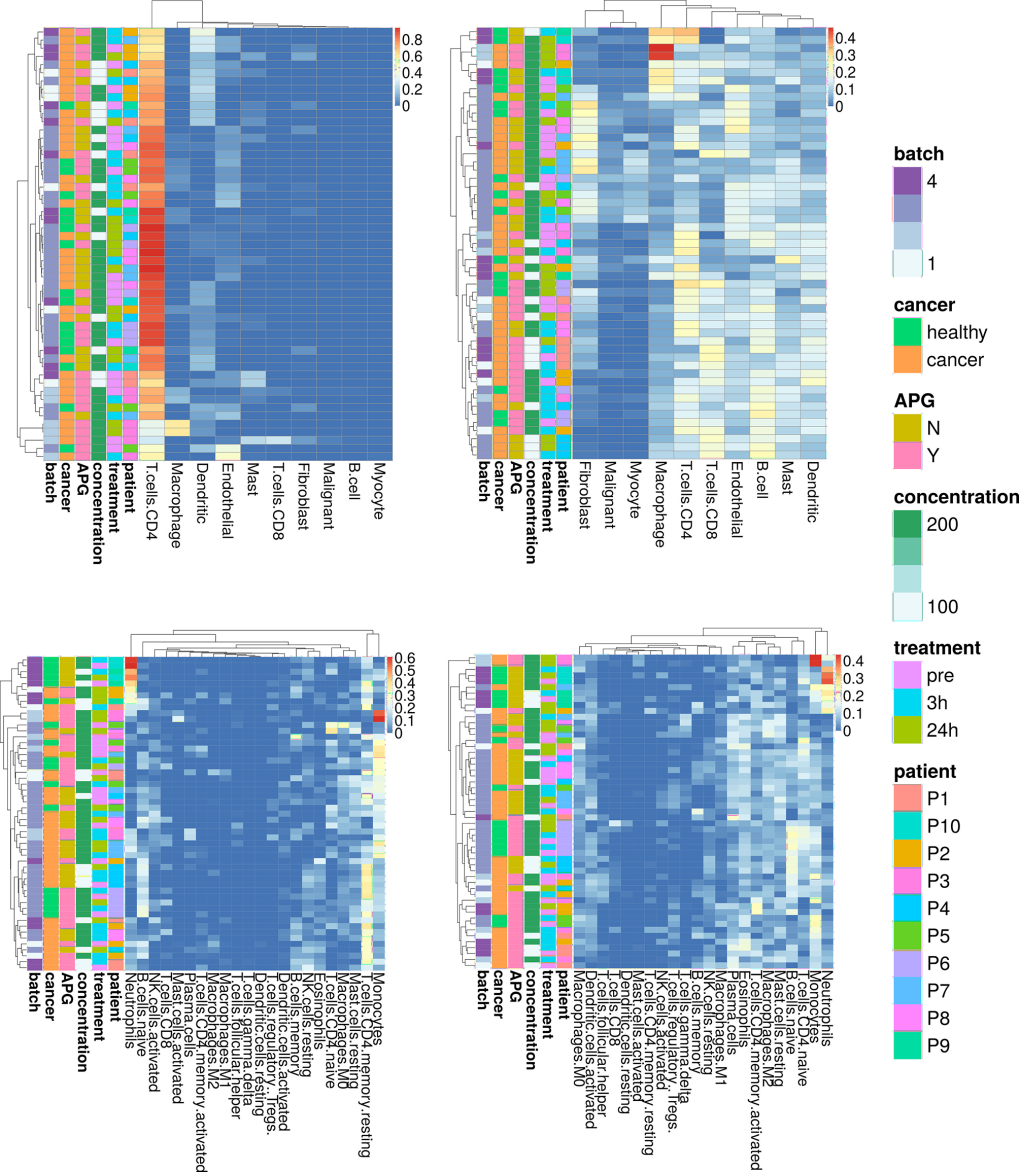
Cell-type deconvolution from plasma cfRNA using two reference datasets and two deconvolution tools. Upper panel: a single cell RNAseq reference dataset from H&N cancer (28). Lower panel contains a reference from lymphocytes LM22 (27). Left panel shows results obtained by CIBERSORTx, right panel results obtained by GEDIT. Results are clustered by proximity. Each row represents a single sample, and an annotation panel is included to the left of each heatmap representing the: sequencing batch (batch), whether the patient has cancer (cancer), whether they received APG-157 (APG), which concentration (concentration), when the sample was collected (treatment) and the blinded patient ID. Some samples contain within batch/across batch technical replicates.

One of the important uses of cfRNA is the ability to inform on the tissue of origin. We performed cell/tissue type deconvolution on plasma cfRNA and estimated the predicted proportion of cell-specific transcripts in each sample. As these predictions are heavily dependent on the choice of algorithm and reference expression matrix, we compared two existing tools, CIBERSORTx (36) and GEDIT (37). We first utilized a single-cell RNAseq dataset from primary and metastatic samples from HNSCC (39). While the tools provided slightly distinct results (**Figure 2**, upper panel), we could conclude that the majority of cfRNA in our study originates from blood and endothelial cells. The presence of malignant cells could not be detected with high confidence. CIBERSORTx was able to predominantly detect CD4+ T-cells, which does not reflect the expected heterogeneity of the circulating transcriptome, while using GEDIT we could detect CD8+ T-cells, endothelial cells and fibroblasts. While none of the cell signatures appeared to be cancer- or treatment-specific, we could observe some patient-specific clustering in the cell proportion estimates, that might hint to the highly individual cfRNA profile dependent on a number of factors.

As the majority of cell types detected in plasma were blood cells, we next used the same approach with a different reference dataset from LM22 (38), consisting of predominantly circulating immune cells (**Figure 2**, lower panel). We observed a higher concordance in predicted cell proportions between the two tools used for the analysis. While neutrophils and monocytes were highly abundant in a subset of samples, we could also detect CD4+ T-cells and B-cells, macrophages and mast cells. We did not detect any significant differences in cell populations between cancer and healthy patients or pre- and post-treatment, possibly due to a large heterogeneity between individuals.

### Differential Expression Analysis

**Figure 3:**
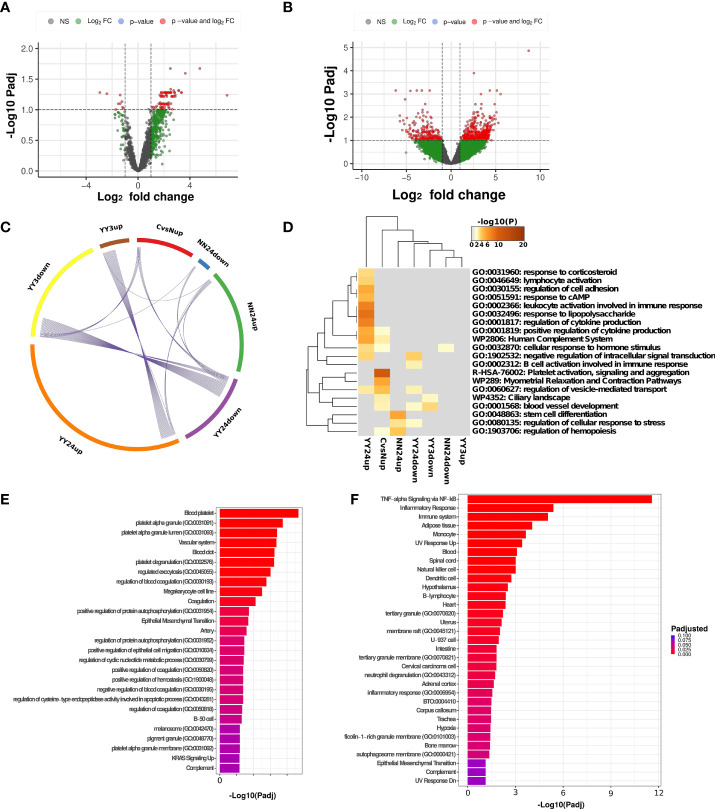
Differential expression analysis. **(A)** Volcano plot representing significantly differentially expressed genes in Cancer vs. Healthy patients at baseline. Genes are considered significant if they pass the FDR-corrected p-value of 0.1 and show an absolute fold difference of at least 2. **(B)** Volcano plot representing significantly differentially expressed genes in APG-treated Cancer patients after 24h vs. baseline. Genes are considered significant if they pass the FDR-corrected p-value of 0.1 and show an absolute fold difference of at least 2. **(C)** Overlap of differentially expressed genes (DEGs) between different comparisons and **(D)** heatmap showing the overlap in overrepresented terms between different comparisons. CvsNup = Cancer vs. Healthy patients before treatment, upregulated genes; YY3up/down APG-treated Cancer patients at 3h post-treatment vs. pre-treatment, up or downregulated genes; YY24up/down APG-treated Cancer patients at 24h post-treatment vs. pre-treatment, up or downregulated genes; NN24up/down Placebo-treated healthy patients at 24h post-treatment vs. pre-treatment, up or downregulated genes. **(E)** Bar plot showing the top most significantly enriched terms in cancer vs. healthy patients at baseline. The dataset is a combination of GO Biological Processes, GO cellular component, MsigDb and Jensen tissues databases. **(F)** Bar plot showing the top most significantly enriched terms in APG-treated cancer patients 24h post-treatment vs. pre-treatment. The dataset is a combination of GO Biological Processes, GO cellular component, MsigDb and Jensen tissues databases.

Differential expression analysis was performed using DESeq2 (32) and taking into account the batch effects in the data. We first set out to find constitutively differentially detected genes in cancer patients compared to healthy controls at baseline, setting a threshold at 2-fold absolute change and an FDR-corrected p-value below 0.1. We found 82 genes uniquely upregulated in cancer patients (**Figure 3A**). We then tested the effects of the treatment after 3 and 24h on the circulating transcriptome. No differences were detected before and after treatment in either placebo-treated cancer patients or APG-treated healthy controls. However, we detected 146 up and 17 downregulated genes after 24h in the placebo-treated control group. It is of note that this group contained only two biological replicates and no technical replicates. Functional enrichment of the 133 upregulated genes did not yield any significant enrichment. We detected 40 up- and 149 down-regulated genes in APG-treated cancer patients after 3h, and 276 and 104 up or downregulated genes, respectively after 24h (**Figure 3B**). Comparing the resulting differentially expressed genes (DEGs) we could find some minor overlaps, mostly in APG-treated cancer patients after 3h and 24h (**Figure 3C**). A small number of genes specific for cancer at baseline were downregulated in APG-treated cancer patients after 3h and 24h (ACVR2B, CACNA1F, DONSON, PIH1D3, PRDM6). **Supplementary Figures 1**, **2** summarize the differentially expressed genes detected in both cancer vs. healthy patients (**Supplementary Figure 1**) and 24h post-APG treated patients (**Supplementary Figure 2**). The data shows a high inter-individual variation but a generally good concordance between technical replicates. These data might indicate a high variability in the circulating transcriptome in different individuals which would warrant an individualized approach in the analysis of such data. The complete list of differentially expressed genes can be found **Supplementary Table 1**.

Using metascape (34) we performed a GOterm enrichment analysis to detect overlaps between multiple datasets mentioned above (**Figure 3D**). Significant associations can be detected in 24h post-APG-treatment in cancer patients and these were likely to be associated with lymphocyte activation and cytokine production. A more detailed enrichment (**Figure 3F**) indeed shows an increase in TNF-alpha associated signaling which might indicate an increase in tumor-associated immune modulation. Some of the notable genes associated with these pathway were FOS, FOSB, FOSL2, all members of the FOS family, as well as TLR2, IL1B, DUSP1, NR4A1 and CCNL1, many of which are proto-oncogens or pro-inflammatory regulators of proliferation and apoptosis. While there was no significant overlap between upregulated DEGs in cancer at baseline and APG-treated cancer patients after 24h, a common term was vesicle-mediated transport, indicating a potential increase in extravesicular trafficking in both conditions. The most striking difference between cancer patients and healthy individuals at baseline was, however, a platelet-associated transcriptional signature (**Figure 3E**). This signature has been already described in the context of metastatic cancer in a previous work (40) and could be a promising diagnostic marker.

## Discussion and Conclusion

The need for minimally invasive disease biomarkers has led to the discovery of circulating tumor cells, tumor DNA, and cell free RNA molecules as potential non-invasive biomarkers (41–43). Advances in sequencing technologies have enabled improved detection of even a small number of circulating tumor cells (CTCs) in blood. Moreover, cell free DNA (cfDNA) has been used to detect driver mutations and epigenetic marker mutations in tumor patients (41, 42). The mutations found in tumor samples, such as the mutation of p16, p53, PI3KCA, Notch1 have also been shown to be detectable in circulating DNA samples (44). Exosomal samples purified from blood have yielded differential expression of miRNAs in early vs late-stage metastatic tumors (43). Taken together, these studies indicate that it is reasonable to attempt to use liquid biopsies for the detection of drug treatment effectiveness on head and neck cancers.

Circulating RNA (cfRNA) shed from tumor cells might be more informative than cfDNA as gene expression levels are more easily interpreted than mutation frequencies. Detection of cfRNA could be carried out from both serum and plasma samples following library construction and sequencing (45). Due to the unique properties of cell stabilization upon collection in the presence of anticoagulants, plasma has been shown to be the material of choice for cfRNA biopsies (46). Here we show an approach for detecting cfRNAs from plasma of cancer patients and healthy individuals without the need for a specialized RNAseq protocol. Employing quality metrics as summarized by Pan W (47). We confirmed little to no DNA contamination and all samples were of sufficient quality for further analysis. In addition to detecting fragmented mRNAs, our results show a number of mRNAs that are covered across their full length and can be detected across all samples, with ACTB as one example. Indeed, Chim et al. (48) have found that ACTB shows a low variability in plasma and could be used as a control gene. Moreover, plasma cfRNAs show little technical variability and can be reliably compared across batches when taking into account the batch effects.

One goal of cfRNA detection is to determine the effectiveness of drug treatment in cancer subjects. Preclinical mouse models and pilot human clinical trials have shown that curcumin treatment could lead to downregulation of cytokine expression. Recently, we have demonstrated in a single oral cancer study that treatment with APG-157 for 3 hours results in the recruitment of immune cells to the tumor site (12). RNA seq and immunofluorescence (IF) investigations showed the presence of CD8 and CD4 positive T cells in the tumor microenvironment (TME). What is not known is the mechanism of T cell recruitment. It is likely that APG-157 treatment leads to immune cell activation from bone marrow stem cells that could be detected using liquid biopsy RNA seq. Thus, in the present investigation, we have performed RNA seq analysis of plasma samples collected from placebo and APG-157 treated head and neck cancer subjects. While the detection of tumor derived RNAs in plasma would be very useful for tumor diagnosis, it has been shown that the presence of these transcripts in plasma of patients can be very limited (49). Indeed, when performing a tissue deconvolution on our samples and using a reference scRNA from a HNC sample including metastatic cells and immune cells, the majority of cell types detected in our samples were immune cell types. This is not unexpected based on the fact that previous studies have found that the majority of transcripts detected in plasma are derived from immune cell types, and a much smaller portion from other cell types. We did not detect a “pathogenic cell type” in this study.

The effects of APG-157 treatment on cancer patients could be observed as changes in plasma RNAs after 24h following treatment. These changes were associated with lymphocyte activation and cytokine production, indicating an immune response and mobilization of immune cells triggered by the treatment. An especially striking observation was the increase of TNF-alpha response which, although contradictory in the response of cancer, could indicate an increase in tumor apoptosis (49, 50). These results also enforce the hypothesis that cfRNAs are not only passive debris coming from dying and dead cells but also active modalities in cell-to-cell signaling (51), and could point to an increased immune cell activation and recruitment. In particular, genes from the FOSL family were increased after 24h treatment with APG-157. These genes encode proteins that can dimerize with proteins of the JUN family and form the transcription factor complex AP-1, a known regulator of cell growth, differentiation proliferation and apoptosis *via* targets such as p53 (52). By contrast, we did not detect an enrichment of tumor-associated pathways.

Our results further show an increased level of platelet associated RNAs in the plasma of HNC patients compared to their healthy counterparts. A similar observation has been made by Beck et al. (40) when comparing lung cancer patients to healthy controls. A role for platelets in cancer metastasis has already been suggested (53–55), and the potential for using platelet-specific RNA signatures as early biomarkers of cancer progression needs to be further examined.

The full characterization of circulating RNAs will require more rigorous studies in the future. These are facilitated by the fact that in the majority of cases, plasma is readily available as a biopsy and it is convenient for multiple sampling when following a patient over a time course. Moreover, RNAs are molecules that, unlike cfDNA, are not only excreted by dead and dying cells but can also inform on active secretion processes from multiple cells in the form of exocytosis (56). While we are currently unable to differentiate between free cfRNAs and those derived from exosomes, we are confident that future technical development will allow for such distinction.

While this small study showed encouraging results and pointed to the potential of using this methodology to monitor the treatment response, a validation study with a larger patient population is necessary in order to verify our findings and help identify individual plasma cfRNA-based biomarkers that could be validated and potentially utilized in clinical practice. Due to the low number of replicates per condition this study might be lacking adequate power to discover a larger number of gene candidates, which should be addressed in replication cohorts. Another limitation of this study is that the ten (10) subjects included in this study were all males. This distribution follows the overall cohort; only one of the cohort was a healthy (non-cancer) female and randomly assigned to the placebo group. Furthermore, while our study showed that it is possible to detect a number of RNAs from as little as 200uL of plasma, further technical advances could reduce the amount of starting material needed. Recently, new technologies such as the SILVERseq (57) have shown promising sequencing results using very small volumes of serum as a liquid biopsy. We therefore believe that cfRNA detection is a valuable tool for the identification of human cancer biomarkers pre and post drug treatments and in conjunction with other data the current study can be a valuable resource for data mining and new biomarker discovery.

## Data Availability Statement

The datasets presented in this study can be found in online repositories. The names of the repository/repositories and accession number(s) can be found below: https://www.ncbi.nlm.nih.gov/geo/query/acc.cgi?acc=GSE196038.

## Ethics Statement

The studies involving human participants were reviewed and approved by Institutional Review Board (IRB) of the Veterans Administration Greater Los Angeles Healthcare System (VAGLAHS) in Los Angeles, California. The patients/participants provided their written informed consent to participate in this study.

## Author Contributions

AT: Conceptualization, data curation, formal analysis, investigation, methodology, validation, visualization, writing–original draft, and writing–review and editing. MM: Data curation, investigation, methodology, validation, and writing–review and editing. SB: Data curation, investigation, writing–original draft, and writing–review and editing. PM: Conceptualization, funding acquisition, project administration, resources, and writing–review and editing. LA: Conceptualization, funding acquisition, project administration, resources, and writing–review and editing. MW: Conceptualization, project administration, supervision, and writing–review and editing. ES: Conceptualization, investigation, methodology, project administration, supervision, validation, writing–original draft, and writing–review and editing. MP: Conceptualization, methodology, supervision, validation, and writing–review and editing. All authors contributed to the article and approved the submitted version.

## Funding

Funding was received from Aveta Biomics Inc and the University of California at Los Angeles Academic Senate as well as the Veterans Administration Greater Los Angeles Healthcare System West Los Angeles surgical education program (to Eri S. Srivatsan and Marilene B. Wang).

## Conflict of Interest

PM and LA are employees of Aveta Biomics Inc, which provided partial funding for the current study, and have a patent pending for Polypharmaceutical Drug Compositions and Related Methods. PM and LA had no role in the recruitment of the subjects and collection and analysis of the samples. All authors were blinded to the study and its results until the study was completed.

The remaining authors declare that the research was conducted in the absence of any commercial or financial relationships that could be construed as a potential conflict of interest.

## Publisher’s Note

All claims expressed in this article are solely those of the authors and do not necessarily represent those of their affiliated organizations, or those of the publisher, the editors and the reviewers. Any product that may be evaluated in this article, or claim that may be made by its manufacturer, is not guaranteed or endorsed by the publisher.

## Supplementary Material

Supplementary Figure 1 Heatmap representing all genes upregulated in cancer vs. healthy patients at baseline. Data are presented as z-scores, and clustering was performed using average linkage.

Supplementary Figure 2 Heatmap representing all genes upregulated in APG-treated cancer patients 24h post-treatment vs. pre-treatment. Data are presented as z-scores, and clustering was performed using average linkage.
